# Accumulation of metals in the leaves of different urban forest tree species and its relation to the proximity to the airport

**DOI:** 10.1007/s11356-026-37427-2

**Published:** 2026-01-21

**Authors:** Evangelia Gkini, Marianthi Tsakaldimi, Ioannis Mousios, Theocharis Chatzistathis, Areti Mpountla, Petros Ganatsas

**Affiliations:** 1 Department of Forestry and Natural Environment, Laboratory of Silviculture, Aristotle University of Thessaloniki, Thessaloniki City, Greece; 2 Hellenic Agricultural Organization “ELGO-DIMITRA”, Institute of Soil and Water Resources, 57001 Thermi City, Greece

**Keywords:** Urban ecology, Air pollution, Metals, Foliar concentrations, Woody species, Evergreens, Deciduous

## Abstract

Metal pollution in urban areas has become a serious problem during the last two decades because of vehicular emission, industrial activity, fossil fuel use, and their accumulation constitutes a serious environmental hazard. The aviation sector puts additional impact on the environment further impacting human health. Urban trees can uptake and accumulate pollutants in their tissues, through their roots and leaves. This study aimed to determine whether airport traffic has toxic effects on airport’s vegetation, to compare five urban trees with different morphological and silvicultural characteristics (*Pinus brutia, Tamarix sp., Populus alba, Olea europaea, Nerium oleander*) regarding their foliar metals (Cu, Ni, Pb, Mn, Fe, Co, Cr, Cd, Zn) accumulation, and to find out how proximity to the airport affects above accumulation. Airport of Thessaloniki, northern Greece (SKG) was the case study where data were collected. Results showed that forest tree species presented different heavy metal accumulation patterns. The metals concentration in leaf samples was low and did not exceed toxicity threshold, both inside and outside the airport area. The taller trees with extensive crown surface area i.e., the deciduous and fast-growing tree species *P. alba* and the evergreen conifer tree species *P. brutia*, were the most affected. The proximity to the airport area had strong influence on the metal's concentrations in the foliage of *P. brutia*, while in the other tree species it significantly affected only one or two metals.

## Introduction

Among the wide range of human activities that can have an impact on the degradation of the natural environment, transportation activity has a fairly important place (Bierza and Palowski 2011; Brtnický et al. 2019; Pollock and St. Clair 2020). Airports, highways, and roads with heavy vehicle traffic are a significant contributor to environmental degradation as they introduce harmful substances into the environment. Metals’ pollution in urban areas has become a serious problem during the last two decades because of transportation (Ganatsas et al. 2011; Zhao et al. 2024), industrial activity, fossil fuel use, and their accumulation constitutes a serious environmental hazard due to their toxicity, persistence and bioaccumulation, which threaten the health of all living organisms and tend to bioaccumulate in the food chain (Huang et al. 2018; El-Khatib et al. 2020; Solomun et al. 2024). Metals are one of the main components of airborne particulate matter (PM2.5) which are related to the increase of respiratory and cardiac diseases (Vαzquez et al. 2016; Zhao et al. 2024). Künzli et al. (2000) report high impact of outdoor (total) and traffic-related air pollution on public health in Austria, France, and Switzerland, which caused 6% of total mortality or more than 40,000 attributable cases per year. About half of this mortality caused was attributed to motorized traffic. They report also, for more than 25000 new cases of chronic bronchitis (adults); more than 290 000 episodes of bronchitis (children); more than 0·5 million asthma attacks; and more than 16 million person days of restricted activities. Also, Carugno et al. (2016) report for at a large regional level (the Lombardy region of Italy), that cause-specific hospitalizations increase with increasing levels of air pollution; specifically, report that 1.0% increase of premature deaths of all natural causes, associated with a 10 μg/m3 increase in the air pollutant level, and quite higher (2.1%) for deaths due to respiratory diseases. Also, report an increase of 5.49% of hospital admissions on cardiac, cerebrovascular and respiratory diseases.

In urban environments, high vegetation (trees) provides many ecosystem services and increases the environmental quality (microclimate improvement, carbon sequestration, noise reduction, soil retention and stormwater drainage, biodiversity, and air pollution mitigation) (Hofman et al. 2016). Urban plants, especially the perennial ones like forest woody species (trees and shrubs), can uptake and accumulate pollutants in their tissues, through their roots and leaves. The removal of dust and air pollutants is one of the important environmental services of trees in urban environments (Bierza and Palowski 2011; Chen et al. 2016; Steinparzer et al. 2023). In particular, urban tree leaves are sensitive and highly exposed to air pollution and have been efficiently used as indicators for heavy metal pollution (Tomašević et al. 2011; Simon et al. 2014; Badamasi 2017; Hrotkó et al. 2021) and thus have a great ecological importance.

Heavy metals can be up taken via roots, or they can be dry-deposited on tree leaf surfaces and accumulated through sedimentation due to gravity. However, each tree species has its own morphological and functional characteristics (e.g. leaf geometry, stomatal size and density, epidermal features, leaf pubescence, tree height, leaf area density, canopy morphology and size, root uptake capacity) that affect its behavior against air pollutants (Simon et al. 2014; Hofman et al. 2016; Santos et al. 2019). For example, trees have a long lifespan and due to their high expansion of crown, occupy a large leaf area and can capture more trace metals compared to shrubs and grasses, (Liang et al. 2017; Hrotkó et al. 2021). Conifers that usually are evergreen, can accumulate higher amounts of pollutants due to their foliage function throughout the entire year, while deciduous tree species shed their leaves every autumn, and thus transfer the yearly input of pollutants into the soil surface with litter fall (Steinparzer et al. 2023).

Although urban forest trees are very often used in air pollution surveys in highly polluted areas, where lichens and mosses are often absent, there are not enough published data on environmental pollution caused by air transport. Air transport is an important part of the global economy and human life quality (Trojanek and Huderek-Glapska 2018; Brtnický et al. 2019). However, the aviation sector has significant environmental impacts and affects human health and other living organisms, not only at a local level, but also at a regional level, or on a global scale (Massas et al. 2018). At a local level (near airports), negative impacts are mainly associated with noise, air, and soil pollution (Ozkurt et al. 2014; Hudda and Fruin 2016; Addepalli et al. 2018; Brtnický et al. 2019). Aircraft-related pollutant sources can be significantly enriched in Pb and Zn but also in Cd, Cu, Ni (Zhao et al. 2024). According to Turgut et al. (2019), Pb, followed by Cd, Cu, Mo, Cr, Ni, Fe, Si, Zn, Na, P, Ca, Mg, and Al are dominant elements that shaped the general aviation aircraft emissions. In the study of Al Khateeb (2017) plants collected from an airport area had the highest Al, Cr, Fe, Cu, Zn, Cd and Pb contents. Respectively, Radomska et al. (2020), found that the greatest soil contamination with metals was observed near the runway. The soil content of heavy metals was found considerable high in manganese, copper, lead, zinc, chromium, and iron as compared with the background soils out of the influence of the airport activities. Massas et al. (2018) investigated heavy metal distribution in soils near Athens International Airport, Greece; their analysis of enrichment factors (EF), availability ratios (AR), and spatial EF patterns indicated ongoing secondary accumulation of metals in the area’s soils.

As there is limited data on metal concentrations in urban trees within airport environments, particularly regarding levels of metals (Cr, Cu, Co, Zn, Ni, Pb, Fe, Mn, Cd) both in areas near the runway and in surrounding areas outside the airport, the purpose of this research was to study the ability of five urban forest trees, with different morphological and silvicultural characteristics (*Pinus brutia, Tamarix sp., Populus alba, Olea europaea* and *Nerium oleander*) to retain heavy metals through their crowns, inside and outside the airport area. Our hypotheses were: a) Leaf concentrations of the following metals Cr, Cu, Co, Zn, Ni, Pb, Fe, Mn, Cd in the above-mentioned tree species are increased within the airport area, b) different morphological and silvicultural characteristics of the urban trees influence the trapping and retention of heavy metals, c) foliage pollution by heavy metals is differentiated between the area inside the airport and the area outside the airport (in a radius of 10 km from airport).

## Materials and methods

### Sampling sites

**Fig. 1:**
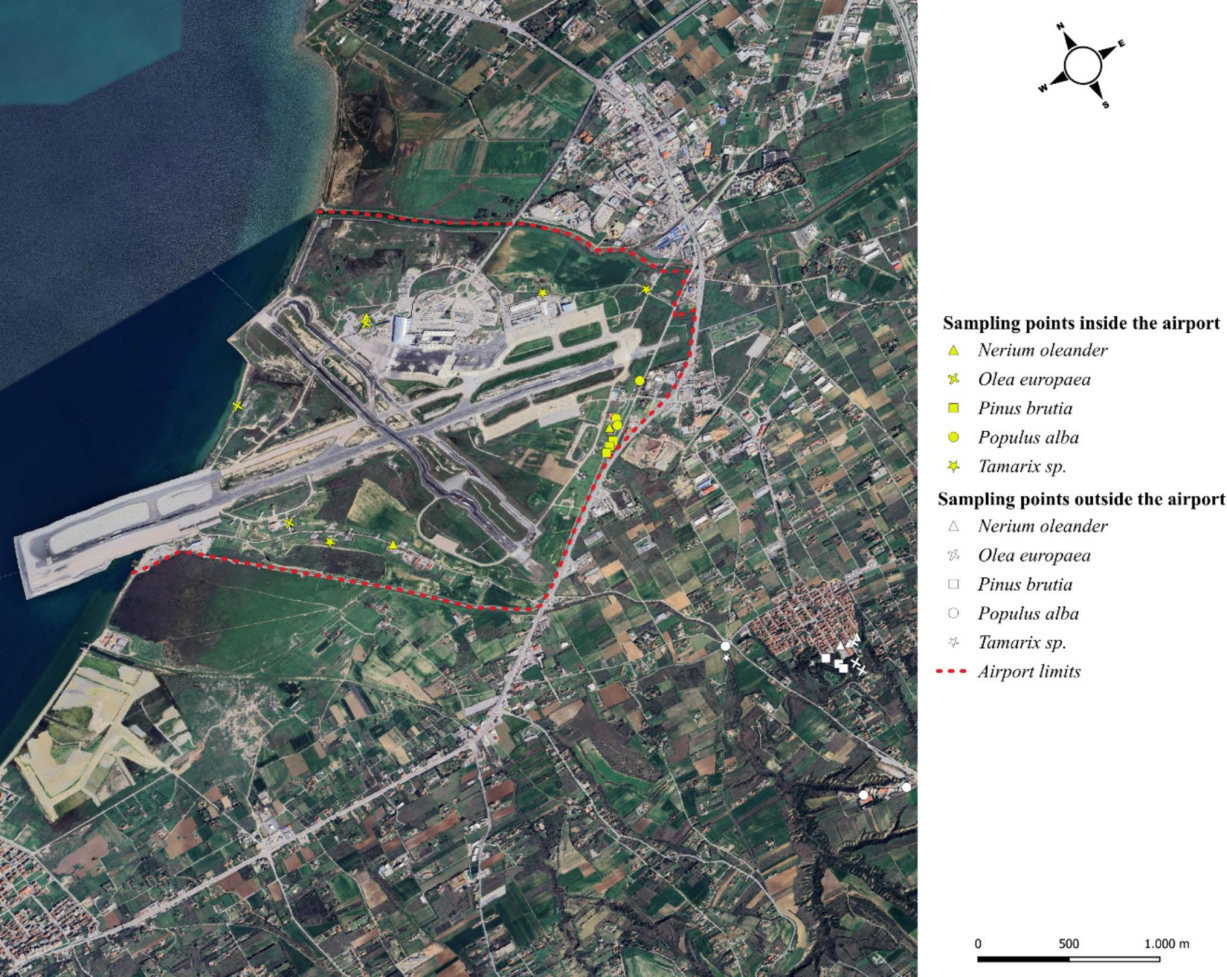
Distribution of sampling points for the urban forest tree species in the airport area; Macedonia Airport Thessaloniki, Greece (Google Earth)

The research was carried out at the international airport area of Thessaloniki (N. Greece), with the official name Macedonia Airport (SKG/LGTS) with latitude 40° 31′ 11" N, longitude 22° 58′ 15" E. It is the only airport serving the metropolitan city Thessaloniki (the second-largest city in Greece) and is located at 16 km from the city center. The area of ​​the airport is 2,306.7 ha; its altitude is 7 m a.s.l. and it has two runways (Fraport Greece 2023a). The annual traffic of the airport for the year 2023 was 7,029,957 million passengers, while in relation to 2022 the percentage of passengers has increased by 18.7% and flights by 11.5%, resulting in approximately 65,000 flights (Fraport Greece 2023b). Sampling sites were taken inside and outside (near) the airport area (Fig. 1). More specifically, outside the airport, the sampling was carried out at selected points located within a radius of ten (10) kilometers from the airport (close to the village Neo Rysio), while their airspace is a runway for aircraft.

### Climatic conditions and vegetation of the study area

The climate can be characterized as Mediterranean, with cold winters and hot temperatures during the summer. According to Meteorological Station “Macedonia” airport of Thessaloniki, the maximum average temperature is 26.6 °C (July) and the minimum average temperature is 5.2 °C (January). The annual mean temperature exceeds 20 °C, the mean relative humidity is about 78.1%, and the annual rainfall is 431.5 mm. The wind direction is unstable following different directions each season. The vegetation of the area belongs to *Quercetalia pubescentis* floristic zone, to the alliance of *Ostryo-Carpinion* (Petaloudi et al. 2022). It hosts many plant species; several of them have been artificially plated (e.g., *Pinus brutia*, *Olea europaea)* and others such as *Tamarix* sp., *Populus alba*, *Nerium oleander* are natural vegetation.

### Field sampling procedure and tree species

The sampling was carried out in the middle of September 2023 over a three-day period, with at least 5 days without rain before sampling (Bierza and Bierza 2024). Our study focused on urban tree species that grow both within the airport area and near it. The collection of leaves and needles was carried out from the following forest woody species *Pinus brutia* Ten*., Tamarix sp., Populus alba* L*., Olea europaea* L*.*, and *Nerium oleander* L*.* as dominant species in the studied area. For each tree species, six (6) individuals, three (3) inside the airport area, and three (3) outside of it and of similar age, were randomly chosen on both sites. The geographic coordinates were recorded for each individual tree and put on a map (Fig. 1). For each individual tree, the following silvicultural characteristics were also recorded: diameter at breast height (dbh) or diameter at trunk base due to short trunk as a shrub (for *Nerium oleander*) (in cm), total tree height (in m), crown length (in m), the diameter of the maximum and minimum axis of the crown (in m), and then the crown surface area (Ca) was calculated (in m^2^). The crown diameters (max and min) were measured from the tree crown projection to the ground, and through two vertical directions. Every length/height was measured with an altimeter. Crown surface area was calculated assuming that the crown is a solid geometric shape (conoid, paraboloid, hemisphere, Eqs. 1, 2, 3 respectively) (Brack 1999) with a measured crown length and crown width (average crown diameter) as follows:

1 $$\mathrm{Conoid}\;\mathrm{crown}\;\mathrm{surface}\;\mathrm{area}\;\mathrm{Ca}\hspace{0.17em}=\hspace{0.17em}(\pi\ast D)/2\ast\surd(L^\wedge\hspace{0.17em}2+\hspace{0.17em}{{(D/2})}^\wedge2)$$

2 $$\mathrm{Paraboloid}\;\mathrm{crown}\;\mathrm{surface}\;\mathrm{area}\;Ca\hspace{0.17em}=\hspace{0.17em}(\pi\ast D)/(12\ast L^\wedge2)\ast(D^\wedge2/4{(+\hspace{0.17em}4L^\wedge2))}^\wedge1.5(-D^\wedge3/8)$$

3 $$\mathrm{Hemisphere}\;\mathrm{crown}\;\mathrm{surface}\;\mathrm{area}\;Ca\hspace{0.17em}=\hspace{0.17em}(\pi\ast D^\wedge2)/2$$

where: D = average crown diameter, L = crown length.

From each individual of the same tree species, fully expanded and healthy leaves were collected from 5 different points on the tree crown (nearby 500 g of leaves), from the same height of the outer crown (at 2 m above soil level), (Samara et al. 2022; Bierza and Bierza 2024; Solomun et al. 2024). Leaf samples covered with soil, dust, or chemical residues, damaged by insects, mechanically injured as well as dead or subjected to moisture or temperature stress, were not selected (Soil and Plant Analysis Council Inc. 1998; Alahabadi et al. 2017; Safari et al. 2018). A total of 75 leaf samples (i.e., 3 individuals × 5 tree species × 5 leaf samples) were collected both inside and outside the airport area (i.e.,150 samples in total). The fresh leaf samples were placed in open, clean plastic bags; in each bag there were five pooled leaf samples from each individual. All bags transferred to the laboratory and then stored at + 4 °C in the dark for analysis (Soil and Plant Analysis Council Inc. 1998; Simon et al. 2014).

### Preparation of samples in the laboratory

All samples preparation were carried out at the Laboratory of Silviculture and the Forest Soil Science Laboratory of the Faculty of Forestry and Natural Environment of the Aristotle University of Thessaloniki (AUTH). The leaf samples from each plastic bag were cleaned (using a soft brush) from any extraneous substances (e.g., soil and dust), and then they left to natural drying. After natural drying, the samples were placed in a ventilated oven (at 80 °C for 24 h), (Jones et al. 1991). After they were dried, they were pulverized using a suitable mill, to ensure homogeneity. Uniform grinding and mixing are a critical process to obtain an accurate result (Soil and Plant Analysis Council Inc. 1998). After grinding and homogenization, a representative sample of the plant material was taken for analysis and storage. Samples were stored under conditions that would minimize deterioration and maintain their integrity; for this reason, airtight plastic storage containers were used.

### Chemical analysis

Lab analyses were performed at the Soil and Water Resources’ Institute, of the Hellenic Agricultural Organization–DIMITRA (ELGO-DIMITRA). Before chemical analysis, the previously dried, ground and weighed leaf tissue samples were prepared for elemental analysis by decomposition/destruction of organic matter. The two methods commonly used are dry ashing (hot temperature combustion) and wet incineration (acid digestion) (Jones et al. 1991). Both methods are based on the oxidation of organic matter using heat and/or acids. In current analyses, the dry ash method was carried out. Dry combustion of 1 g of dried and ground leaf tissue was performed in a porcelain capsule. The capsules were placed in an annealing oven at 550 °C for 5 h. After 4 h, the samples were returned to ambient temperature. Then, for each sample, 5 ml of 6 N HCl was taken and made up to 50 ml with distilled water. Each sample was then filtered. Finally, an inductively coupled plasma spectrometer (ICP, Optical Emission Spectrometer Avio 220 Max) was used to determine the concentration of metals in the filtrate (diluted ash). The respective detection wavelengths used and the lowest detectable metal value, according to the instrument specifications were (Table 1):

**Table 1:** Wavelength used to detect the mentioned metals and their minimum traceable limits in ppm using the ICP, Optical Emission Spectrometer Avio 220 Max

Heavy Metal	Wavelength (mm)	Minimum traceable limit (ppm)
Manganese (Mg)	257.610	0.0014
Zinc (Zn)	206.200	0.0059
Iron (Fe)	238.204	0.0046
Copper (Cu)	327.393	0.0097
Cadmium (Cd)	228.802	0.0027
Cobalt (Co)	228.616	0.007
Chromium (Cr)	267.716	0.0071
Nickel (Ni)	231.604	0.015
Lead (Pb)	220.353	0.042

### Statistical analysis

All statistical analyses were performed using SPSS software version 29, with significance level set at *p* < 0.05. To test whether the average concentrations of metals in leaf samples differ between the five urban forest trees, and as well as between inside and outside the airport area, the analysis of variance (ANOVA) was applied, and the Duncan's multiple range test was performed for means comparisons. Kolmogorov–Smirnov test was used to determine the normality of data distribution. The homogeneity of variances was tested with Levene's test.

## Results

### Silvicultural characteristics of the studied urban tree species

The main silvicultural characteristics of the studied tree species are presented in Table 2. Αs expected, the selected tree species, four evergreens (two of which have needle-like leaves) and one broadleaved, present quite different morphological idiosyncrasy and functional traits. Individuals of *P. alba* and *P. brutia*, as being tall trees, present the largest height, dbh and crown surface area, while the rest species follow accordingly. The tree crown surface area reflects the available area for leaves to capture atmospheric pollution. The distance of the crown from the ground was also greater for *P. brutia* and *P. alba* trees in relation to *Tamarix*, *O. europaea* and *N. oleander*.

**Table 2:** The main silvicultural characteristics of the studied urban forest tree species. Values are the means with the corresponding standard error of the mean in parenthesis

Tree species	Diameter (dbh/ground base) (cm)	Total tree height (m)	Trunk height (m)	Crown Length (m)	Diameter at maximum axis of the crown (m)	Diameter at minimum axis of the crown (m)	Crown Surface area (Ca) (m^2^)
*Pinus brutia*	45.10 (3.57)	10.47 (0.78)	3.70 (0.42)	6.77 (0.73)	9.27 (0.44)	5.33 (0.33)	88.15 (4.58)
*Populus alba*	36.70 (5.66)	11.30 (1.19)	1.60 (0.14)	9.70 (0.49)	7.27 (0.49)	3.67 (0.61)	93.94 (6.42)
*Tamarix sp.*	9.83 (0.84)	2. 63 (0.29)	0.31 (0.09)	2.32 (0.34)	4.83 (0.37)	2.43 (0.38)	15.85 (1.24)
*Olea* *europaea*	16.77 (0.90)	5.57 (0.26)	2.74 (0.26)	2.83 (0.35)	2.80 (0.63)	1.47 (0.35)	7.16 (1.22)
*Nerium oleander*	5.63 (0.26)	2.07 (0.08)	0.17 (0.04)	1.90 (0.11)	2.13 (0.18)	1.43 (0.14)	4.97 (0.43)

### Metals retention in leaves of different urban forest tree species

Statistical analysis showed that metals capture by foliage varied considerably among urban tree species, both within the airport area and outside it (Tables 3 and 4). The studied metals were found in quite low concentrations, presented different trends, and did not follow a standard pattern in leaf samples.

**Table 3:** Comparison of metals’ concentrations (mg kg^−1^) among the studied tree species, inside the airport area. Within the same metal, values followed by different letters are significantly different (p < 0.05, Duncan's test)

	Mean concentration (Std error of mean in parenthesis), in mg kg^−1^ dry matter								
Urban Forest Trees	Cr	Cu	Co	Zn	Ni	Pb	Fe	Mn	Cd
*Pinus brutia*	1.481 a (0.111)	5.542 (0.182)	0.092 bc (0.006)	20.773 b (1.466)	1.164 a (0.087)	1.155 a (0.407)	227.533 a (20.914)	21.743 b (2.457)	0.009 c (0.006)
*Populus alba*	0.309 b (0.014)	5.334 (0.559)	0.728 a (0.083)	55.463 a (1.755)	1.094 a (0.256)	0.062 b (0.013)	62.093 c (5.307)	30.287 ab (2.774)	0.030 b (0.013)
*Tamarix sp.*	0.499 b (0.097)	6.345 (0.416)	0.171 b (0.066)	13.149 c (1.973)	0.495 b (0.068)	0.268 b (0.127)	167.166 b (14.042)	32.400 a (3.176)	0.330 a (0.247)
*Olea europaea*	0.398 b (0.059)	5.427 (0.639)	0.019 bc (0.007)	15.046 c (1.291)	0.779 ab (0.200)	0.133 b (0.075)	97.410 c (12.252)	23.163 ab (2.786)	0.003 c (0.000)
*Nerium oleander*	0.418 b (0.009)	6.609 (0.597)	0.006 c (0.000)	18.070 bc (1.149)	0.958 ab (0.014)	0.062 b (0.013)	87.840 c (5.672)	28.123 ab (3.563)	0.003 c (0.000)

**Table 4:** Comparison of metals’ concentrations (mg kg^−1^) among the studied tree species outside the airport area. Within the same metal, values followed by different letters are significantly different (p < 0.05, Duncan's test)

	Mean concentration (Std error of mean in parenthesis), in mg kg^−1^ dry matter								
Urban Forest Trees	Cr	Cu	Co	Zn	Ni	Pb	Fe	Mn	Cd
*Pinus brutia*	0.937 a (0.057)	4.089 b (0.189)	0.043 b (0.004)	13.330 b (0.230)	0.725 b (0.028)	0.116 (0.075)	103.533 b (6.272)	25.383 cd (2.422)	0.003 b (0.000)
*Populus alba*	0.647 b (0.149)	5.582 ab (0.928)	0.608 a (0.173)	19.847 a (2.770)	1.382 a (0.107)	0.061 (0.019)	77.557 c (6.709)	53.910 a (6.842)	0.003 b (0.000)
*Tamarix sp.*	0.499 bc (0.024)	5.720 ab (0.354)	0.155 b (0.008)	11.857 b (0.223)	0.609 b (0.042)	0.266 (0.051)	177.933 a (8.096)	36.663 bc (0.292)	0.076 a (0.008)
*Olea europaea*	0.260 c (0.011)	4.843 b (0.585)	0.006 b (0.000)	12.117 b (0.362)	0.699 b (0.189)	0.269 (0.120)	60.617 c (0.689)	20.567 d (1.443)	0.003 b (0.000)
*Nerium oleander*	0.349 c (0.700)	7.368 a (0.453)	0.037 b (0.027)	15.173 b (0.826)	1.007 ab (0.291)	0.271 (0.166)	71.093 c (0.721)	46.437 ab (4.518)	0.003 b (0.000)

The urban tree species most affected by pollution inside the airport area, exhibiting the highest concentrations of most metals, were *P. brutia*, *P. alba*, and *Tamarix* sp., followed by the species *N. oleander* and *O. europaea*. More specifically, inside the airport area (Table 3), the highest foliar Cr concentration was detected in *P. brutia* needles, which was significantly higher (p < 0.05) than that of the other four tree species. The highest foliar Pb and Fe concentrations were also detected in *P. brutia*. Concentrations of Co and Zn were significantly greater in *P. alba*. *Tamarix* sp. needles retained significantly higher Cd concentration than the other species. Ni concentrations were found significantly increased in foliage of the *P. brutia* and *P. alba,* but they did not differ with those detected in *O. europaea*. While, the highest foliar Mn concentration was observed in *Tamarix* sp.; however, it did not significantly differ from those of *P. alba*, *O. europaea*. Mean foliar Cu concentrations slightly varied among the tree species (p > 0.05).

Outside the airport area (Table 4), the highest Cr concentration was also found in the *P. brutia* needles. *N. oleander* leaves had the highest Cu concentration, while it did not significantly differ from that of *P. alba* and *Tamarix*. Co and Zn concentrations remained significantly greater in *P. alba*. Significantly high Ni concentrations were only found in *P. alba* and *N. oleander*. Foliar Pb accumulation did not show significant differences between tree species (p > 0.05)*.* Significantly high Fe and Cd concentrations were detected in *Tamarix.* Higher Mn concentration was found in the leaves of *P. alba,* while did not significantly differ with that of *N. oleander*.

### The differentiation of foliage pollution by metals between inside and outside the airport area

**Fig. 2:**
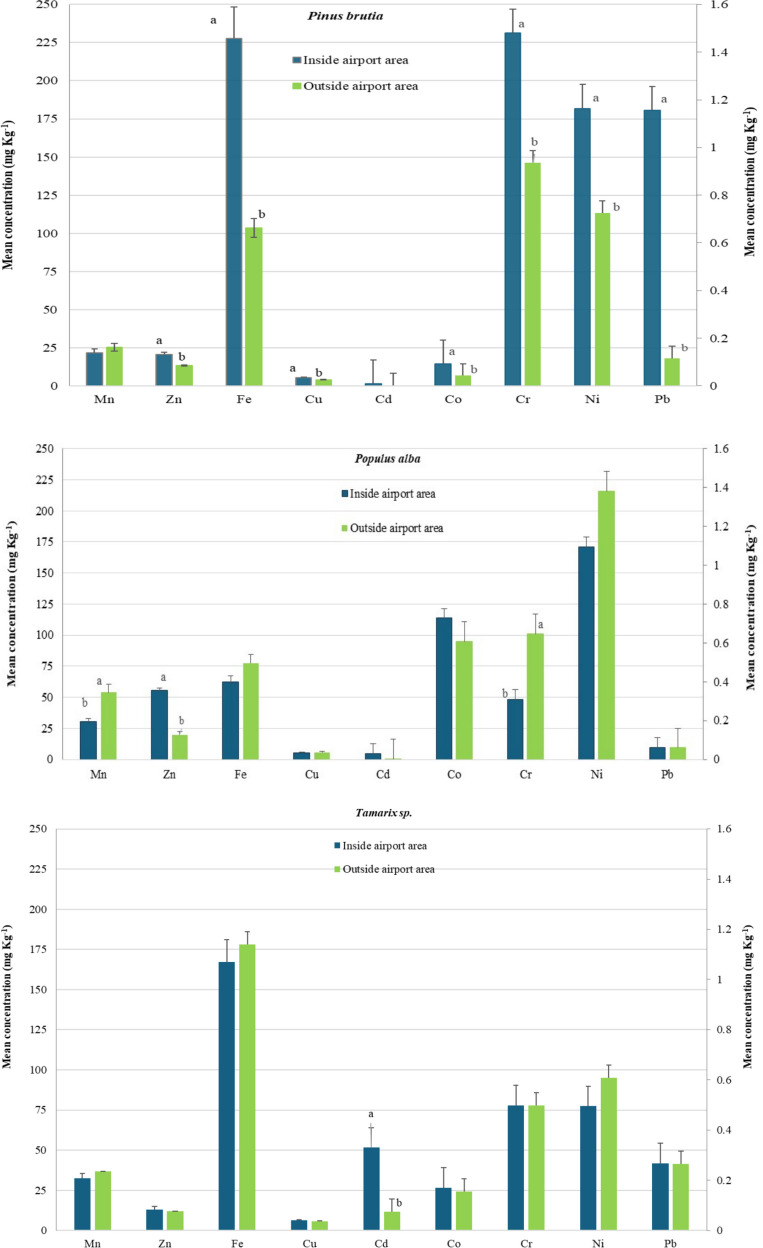

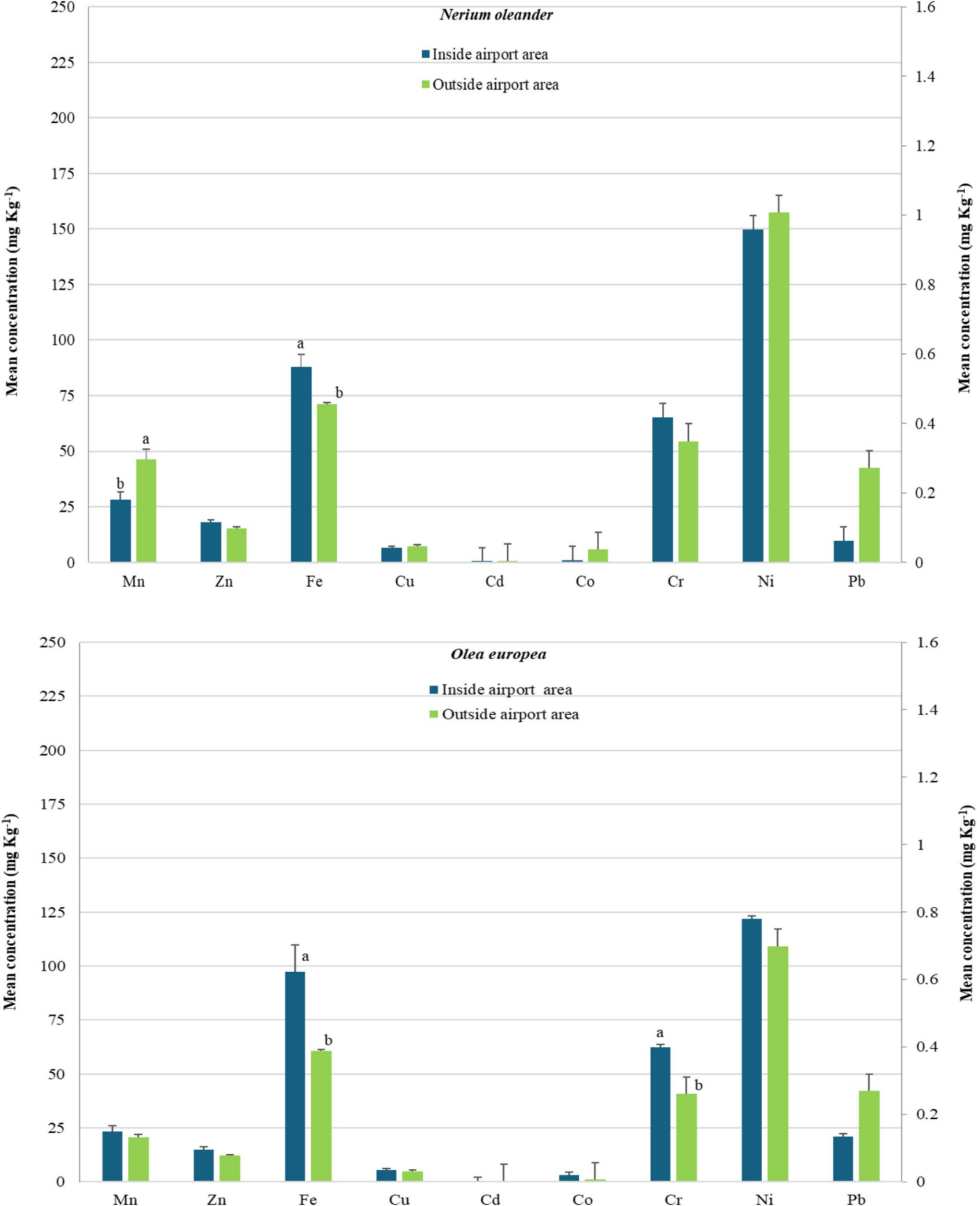
Foliar metals’ concentrations (error bars show the standard error of the mean) of the studied urban forest tree species, in mg kg^−1^. The concentrations of Mn, Zn, Fe, and Cu are represented on the left Y-axis, whereas those of Cd, Co, Cr, Ni, and Pb are represented on the right Y-axis Within the same metal, values followed by a different letter (a or b), denote significant differences (p < 0.05) between inside and outside airport area

Τhe distance of the urban trees from the pollution source (airport infrastructure) played a vital role in their foliar metal concentrations (Fig. 2). Inside the airport area, foliar concentrations of Cr, Cu, Co, Zn, Ni, Pb, and Fe in *P. brutia* trees were found to be significantly higher (p < 0.05) compared to those of trees located outside. A similar pattern was observed in *O. europaea* for Cr and Fe, in *P. alba* for Zn, in *Tamarix* sp. for Cd, and in *N. oleander* for Fe. Conversely, outside the airport area, leaves of *P. alba* exhibited significantly higher accumulations of Cr and Mn, and *N. oleander* leaves exhibited significantly higher Mn concentrations compared to trees located inside the airport area.

## Discussion

The results of this study demonstrate that foliar metal accumulations varied markedly among the studied urban forest tree species, both inside and outside the airport area. These accumulations reflect the cumulative impact of the airport’s daily operations and associated infrastructure, as well as vehicular traffic and overall pollution in the broader airport region.

Although concentrations of the studied metals were relatively low, species-specific differences were evident, and no uniform pattern of accumulation was observed across all leaf samples. Foliar metals’ concentrations of Cr, Cu, Co, Zn, Ni, Pb, Fe, Mn, Cd, were found within normal limits, and quite a bit less compared to the reported critical (toxic) values, both inside airport area and outside it (Påhlsson 1989; Kabata-Pendias and Pendias 2001; Madejón et al. 2004; Solomun et al. 2024). Many of these metals such as Fe, Cu, Zn, Mn are essential nutrients for trees, and are required by various enzymes, whereas others such as Cd and Pb, have no physiological function in trees, and can be toxic if present in high concentrations. For example, in the present study, the recorded foliar metals concentrations in *P. alba* were considerably lower at both sites, than those reported for white poplar trees grown on the banks of a contaminated river in Spain, ​where some of metals reached phytotoxic levels (e.g. maximum values of Cd 15.4 mg kg^−1^, Zn 1312 mg kg^−1^, Fe 814 mg kg^−1^) (Madejón et al. 2004). However, it is worth noting that in the study by Madejón et al., in addition to the different pollution sources, a different sampling and processing method was applied, as the leaf samples were collected from a height of 5 m and washed with a solution. Similarly, foliar metal concentrations in *P. brutia* needles were lower or between those recorded in *Pinus nigra* and *Pinus sylvestris* in a botanical garden in Slovakia (e.g. Cd > 0.15 mg kg^−1^, Ni > 6.5 mg kg^−1^, Zn > 46 mg kg^−1^, Fe > 118 mg kg^−1^, Cu > 4 mg kg^−1^) whose needles were washed with deionized water (Parzych et al. 2017). Likewise, foliar metal concentrations in *Tamarix* sp., *O. europaea* and *N. oleander* were much lower than values reported in other studies, both in polluted and non-polluted areas (Dongarrà et al. 2003; Espinosa and Oliva 2006; Blanusa et al. 2015; Santos et al. 2019; Jeddi et al. 2021; Chatzistathis et al. 2022). Metal concentrations in foliage depend, among other factors, on plant species and genotypes, particularly on morphological and functional traits. The results of this study support the notion that woody species differ in their heavy metal deposition capacity (Tables 3 and 4, Fig. 2), attributable to their characteristics such as leaf’s form, longevity and surface traits, crown surface area, crown form etc., (Simon et al. 2014; Liang et al. 2017). Although evergreen species are generally reported to retain higher foliar metal concentrations than deciduous ones, other studies suggest that broadleaves play also an important role in removing airborne pollutants (Sawidis et al. 2011; Liang et al. 2017; Ianovici et al. 2020), and among broadleaves, those with rough leaf surfaces and trichomes are more efficient than those with smooth ones (Sæbø et al. 2012; Simon et al. 2014). However, foliar metal allocation depends not only on tree characteristics (e.g. leaf and crown traits), but also on metal behavior, solubility and bioavailability in soils (Chatzistathis et al. 2015; Chenchen et al. 2019).

Foliar metal concentrations exhibited site-specific patterns. Inside the airport area, among the tree species investigated, *P. brutia*, *P. alba*, and *Tamarix* sp. were most affected, consistently exhibiting the highest concentrations for most metals (Table 3). It should be noted, however, that the existed *P. brutia* and *P. alba* trees were also located near the regional road (Fig. 1), which may have influenced their foliar metal concentrations. Secondary responses with lower accumulation were recorded in *N. oleander* and *O. europaea* trees.

Regardless of location, inside or outside the airport (Tables 3 and 4 and Fig. 2), the evergreen conifer *P. brutia* retained significantly high foliar concentrations of Cr both inside and outside the airport area, and Ni, Pb and Fe only inside. For the fast-growing deciduous *P. alba*, significantly higher foliar concentrations of Ni, Co, Zn and Mn were detected in the most polluted site (inside the airport area), and Cu, Ni, Mn, Co, and Zn were detected outside the airport. Both species, although they have different morphological and functional traits, are both characterized by much greater crown surface area in relation to other studied tree species (Table 2). The high foliar metal accumulation in *P. brutia* could be explained by the long-lived needles (which remain in the tree from 2–5 years), with rough surface and epicuticular wax that enhance trapping and absorption of air pollutants (Samara et al. 2022). The presence of resins or honeydew deposits from aphids make the needles sticky, and increase dust retention (Sawidis et al. 2011; Saebo et al., 2012; Sawidis et al. 2012; Alahabadi et al. 2017; Liang et al. 2017). In *P. alba,* high foliar metal allocation could be attributed to the large size of leaf surface, which enhances capture of airborne particles captured and associated trace elements (Simon et al. 2014). Foliar metal concentrations in *Tamarix* sp. also displayed site-specific patterns (Tables 3 and 4); Mn concentration was significantly elevated inside airport area and was not statistically different from that of *P. alba*, Fe was highest outside the airport, and Cd was consistently statistically higher at both sites. These patterns may be explained by the species’ small rough needle-shaped leaves that ensheath the wiry twigs, enhancing pollutant capture and metal retention (Sawidis et al. 2011; Alahabadi et al. 2017; Liang et al. 2017). Concerning *N. oleander*, significantly high foliar Ni and Mn concentrations were observed at both sites. This species’ leaves with numerous stomata, thick cuticle covered by a layer of mucilage and hairs, facilitate pollutant trapping (Sawidis et al. 1995; Espinosa and Oliva 2006). Vázquez et al. (2016) also concluded that *N. oleander* plants are a suitable tool to assess airborne metal deposition, especially for Pb. Nevertheless, Ni and Mn are essential micronutrients for plant metabolism and possess active uptake pathways. Their accumulation in foliar tissues may therefore predominantly reflect root uptake from soils and subsequent translocation to leaves, rather than selective retention of particulate matter on leaf surfaces. The existed trees of *O. europaea* inside the airport area showed significantly higher foliar concentrations of Cr and Fe, but overall, *Olea* trees retained the lowest foliar metal concentrations compared to the other urban tree species. This aligns with findings by Blanusa et al. (2015), showing *O. europaea* leaves accumulate less metals after exposure to traffic among the other species sharing similar characteristics, e.g. sclerophylly, longevity, pronounced waxes, roughness. The lowest foliar metals concentrations may be influenced by the location of the sampled *Olea* trees, since these were on the sites furthest from the main airport infrastructure and close to sea front.

The “Macedonia” airport environment significantly influenced foliar metal accumulation compared to the area outside it. Potential metal sources include aircraft emissions, liquid fuels, diesel, and condensed hydro-carbonates particulate matter (PM) generated during combustion processes caused by motor vehicles traffic emissions, road runoff, transportation-related activities (due to the wear of tires, brakes, and roads), (Massas et al. 2018; Zhao et al. 2024; Vázquez et al. 2016). Thus, for the majority of the trees studied, statistically significant differences in foliar metal concentrations were observed between inside and outside the airport area. The proximity to the airport infrastructure and activities significantly influenced foliar Cr, Cu, Co, Zn, Ni, Pb and Fe concentrations in *P. brutia,* while in the rest tree species, only one or two metals were affected; Cr and Fe (in *O. europaea*), Zn (in *P. alba*), Cd (in *Tamarix*), and Fe (in *N. oleander*). Of particular interest, Pb in *P. brutia* needles and Cd in *Tamarix* needle-shaped leaves, inside the airport area were 11.7 and 4 times higher, respectively, than outside. Similarly, other studies showed that plants collected close to airport had the highest foliar metal contents (Al Khateeb 2017; Radomska et. al. 2020). According to Turgut et al. (2019), foliar concentrations decrease as the distance from aircraft emissions source increases.

## Conclusions

According to the findings of this research, it can be concluded that the metals (Cr, Cu, Co, Zn, Ni, Pb, Fe, Mn, Cd) allocation in leaves of all urban tree species studied, were low, within the permissible limits, and no toxic concentrations were detected, both inside and outside the airport area. Foliar metal concentrations varied among the five evergreen and broadleaf urban trees (*P. brutia, P. alba, Tamarix sp. O. europaea, N. oleander*), common in many Mediterranean urban environments, primarily reflecting leaf and silvicultural characteristics (e.g. leaf traits, longevity, crown length and surface area, etc.) rather than microenvironmental factors (e.g. wind direction was quite similar), indicating that diverse vegetation is able to provide a wide range of ecological services. Inside the airport area, the taller trees with extensive crown surface area i.e., the deciduous and fast-growing tree species *P. alba,* and the evergreen conifer tree species *P. brutia,* and also the evergreen *Tamarix* sp. with needle-shaped leaves were most affected, as they retained the highest foliar metals’ concentrations compared to other tree species. The proximity to the airport infrastructure had significant and strong influence on the *P*. *brutia* foliar Cr, Cu, Co, Zn, Ni, Pb and Fe concentrations, while other species were affected to a lesser extent. These findings provide a basis for selecting greening and air-quality improving tree species and highlight the potential of appropriate tree species selection to enhance atmospheric cleaning and urban ecological services. While further insights into metal concentrations and flow are necessary to strengthen the results of the present study, which could furthermore serve as a baseline data for future environmental impact assessment.

## Data Availability

Data supporting the results reported in the article can be found in Tables and Figures included in this paper.
