# Correction to: Accumulation of metals in the leaves of different urban forest tree species and its relation to the proximity to the airport

**DOI:** 10.1007/s11356-026-37720-0

**Published:** 2026-04-02

**Authors:** Evangelia Gkini, Marianthi Tsakaldimi, Ioannis Mousios, Theocharis Chatzistathis, Areti Mpountla, Petros Ganatsas

**Affiliations:** 1 Department of Forestry and Natural Environment, Laboratory of Silviculture, Aristotle University of Thessaloniki, Thessaloniki City, Greece; 2 Hellenic Agricultural Organization “ELGO-DIMITRA”, Institute of Soil and Water Resources, 57001 Thermi City, Greece

**Correction to: Environmental Science and Pollution Research (2026) 33:1353-1364**

10.1007/s11356-026-37427-2

The 1^st^ author given name is Evangelia not Evaggelia.

The Original article has been corrected.

